# Versatile carbon-loaded shellac ink for disposable printed electronics

**DOI:** 10.1038/s41598-021-03075-4

**Published:** 2021-12-10

**Authors:** Alexandre Poulin, Xavier Aeby, Gilberto Siqueira, Gustav Nyström

**Affiliations:** 1 Cellulose and Wood Materials Laboratory, EMPA, Swiss Federal Laboratories for Materials Science and Technology, 8600 Dübendorf, Switzerland; 2 Department of Health Sciences and Technology, ETH Zurich, 8092 Zurich, Switzerland

**Keywords:** Electronic devices, Electrical and electronic engineering

## Abstract

Emerging technologies such as smart packaging are shifting the requirements on electronic components, notably regarding service life, which counts in days instead of years. As a result, standard materials are often not adapted due to economic, environmental or manufacturing considerations. For instance, the use of metal conductive tracks in disposable electronics is a waste of valuable resources and their accumulation in landfills is an environmental concern. In this work, we report a conductive ink made of carbon particles dispersed in a solution of shellac. This natural and water-insoluble resin works as a binder, favourably replacing petroleum-derived polymers. The carbon particles provide electrical conductivity and act as a rheology modifier, creating a printable shear-thinning gel. The ink’s conductivity and sheet resistance are 1000 S m^−1^ and 15 Ω sq^−1^, respectively, and remain stable towards moisture. We show that the ink is compatible with several industry-relevant patterning methods such as screen-printing and robocasting, and demonstrate a minimum feature size of 200 μm. As a proof-of-concept, a resistor and a capacitor are printed and used as deformation and proximity sensors, respectively.

## Introduction

Driven by recent advances in the field of printed electronics^1^ and the expanding Internet of Things (IoT) ecosystem^2^, disposable electronics is emerging as a new class of devices. The integration of electronics in disposable and short-lived goods such as smart packaging^3^ shifts the requirements on electronic components, notably on service life, which counts in days instead of years. Considering the exponentially growing number of IoT devices and the environmental threat that electronic waste represent^4–6^, there is an imperative need for new materials that strike a balance between electronic performance, cost, manufacturability and sustainability.

Standard electronic materials are often not adapted for disposable electronics due to economic, environmental or manufacturing considerations. As a result, significant efforts have been made to develop electrically conductive inks compatible with additive manufacturing techniques and enabling low-cost high volume fabrication of printed circuitry. Available inks are often based on metals^7^, which come in the form of nanoparticles^8^, nanowires^9^ or precursors^10^. Photonic flash sintering^11^ or chemical sintering^12,13^ allows metal-based inks to be processed on inexpensive and flexible substrates. Whereas metals maximize electrical performance, reaching bulk conductivities of around 10^7^ S m^−1^, it represents a waste of valuable resources as well as an environmental concern if integrated in disposable technologies.

Various metal-free inks have also been developed based on electroceramics^14–16^ and intrinsically conductive polymers (ICPs)^17–20^. Indium tin oxide (ITO) is the most prominent electroceramic. It is widely used in devices where optical transparency of the electrodes is required, it shows bulk electronic conductivities of around 10^6^ S m^−1^ and is moisture stable. However, the need for post-deposition annealing, brittleness and high cost of ITO restrict its application for printed electronics. ICPs can provide optical transparency at lower cost and without the need for annealing. However, whereas they can reach bulk conductivities of around 10^6^ S m^−1^, they are often unstable towards atmospheric moisture. Moreover, the general brittleness and limited solubility of ICPs restrict their application for printed electronics.

Carbon is arguably the most widely used material for the development of metal-free electrodes^21–23^. Its different forms can be used individually or in combination, including carbon black, graphene, graphite and nanotubes. Carbon is inexpensive, non-toxic, as well as moisture, pH and temperature stable. It can provide high electrical performance, with graphene basal plane exhibiting electrical conductivity of around 10^5^ S m^−1^^24,25^. Furthermore, it is a naturally occurring and abundant resource that can also be produced from renewable resources^26^. Carbon materials are typically combined with a binder to form composite inks^27^. The use of a binder can improve the mechanical properties of the ink through enhanced carbon–carbon and carbon-substrate interactions, as well as enable 3D printing by reaching higher solid content. However, binders typically being the dielectric phase of the composite, can also limit the electrical properties in terms of resistivity and temperature stability. A careful optimization of the binder to filler ratio is therefore required in the development of conductive composite inks.

A wide range of polymeric binders have been used for printed electronics^28,29^. Common options include acrylic^30^, silicone^31^, styrene^32^, fluoroelastomer^33^ and polyurethane materials^34^. The choice of the binder mainly depends on the properties of the filler and the intended application, which can require features such as self-healing, water stability, heat stability or stretchability. Biodegradable options include poly(lactic acid) (PLA)^35^, poly(vinyl alcohol)^36^, polyurethane (PU)^37^, silk fibroin^38^ and cellulose^39^. The only ones to provide stability towards water, namely PLA and PU, are also the most difficult to decompose in natural conditions and typically require industrial composting infrastructures^40,41^.

Here, we developed an electrically conductive ink composed of carbon particles dispersed in shellac. This natural resin acts as a renewable, biodegradable^42^ and water-insoluble binder between the electrically conductive carbon particles. Shellac already finds a wide range of commercial applications that range from nail polish to edible coatings in the food (E 904) and pharmaceutical industries. However, its use in printed electronics remains limited and the rare references to carbon-loaded shellac electrodes focus on system-level performance rather than ink development^43,44^. Here, we focus on the optimization of the carbon-loaded shellac system. The resulting ink demonstrates low sheet resistance (15 Ω sq^−1^), mechanical flexibility, stability towards moisture and a versatile rheology compatible with a wide range of 2D and 3D additive manufacturing techniques.

The ink formulation, printing techniques and characterization protocols are detailed in "Experimental methods".﻿ The design requirements, materials properties and printability of the ink are presented in “Results and discussion”, as well as proof-of-concept printed proximity and deformation sensors.

## Results and discussion

### Ink requirements and optimization

The transition towards disposable and short-lived electronics comes with several important challenges, including the need for more sustainable materials. As central requirements of sustainability in the development of our electrically conductive ink, we defined that it should be metal-free, as well as exclusively composed of biodegradable or non-toxic materials. We also defined performance and manufacturability requirements, namely that the ink should provide electrical conductivity above 100 S m^−1^ (based on the performance of reported carbon composites for additive manufacturing^45^), stability towards moisture and moderate heat, mechanical flexibility and compatibility with 2D and 3D printing technologies.

**Figure 1:**
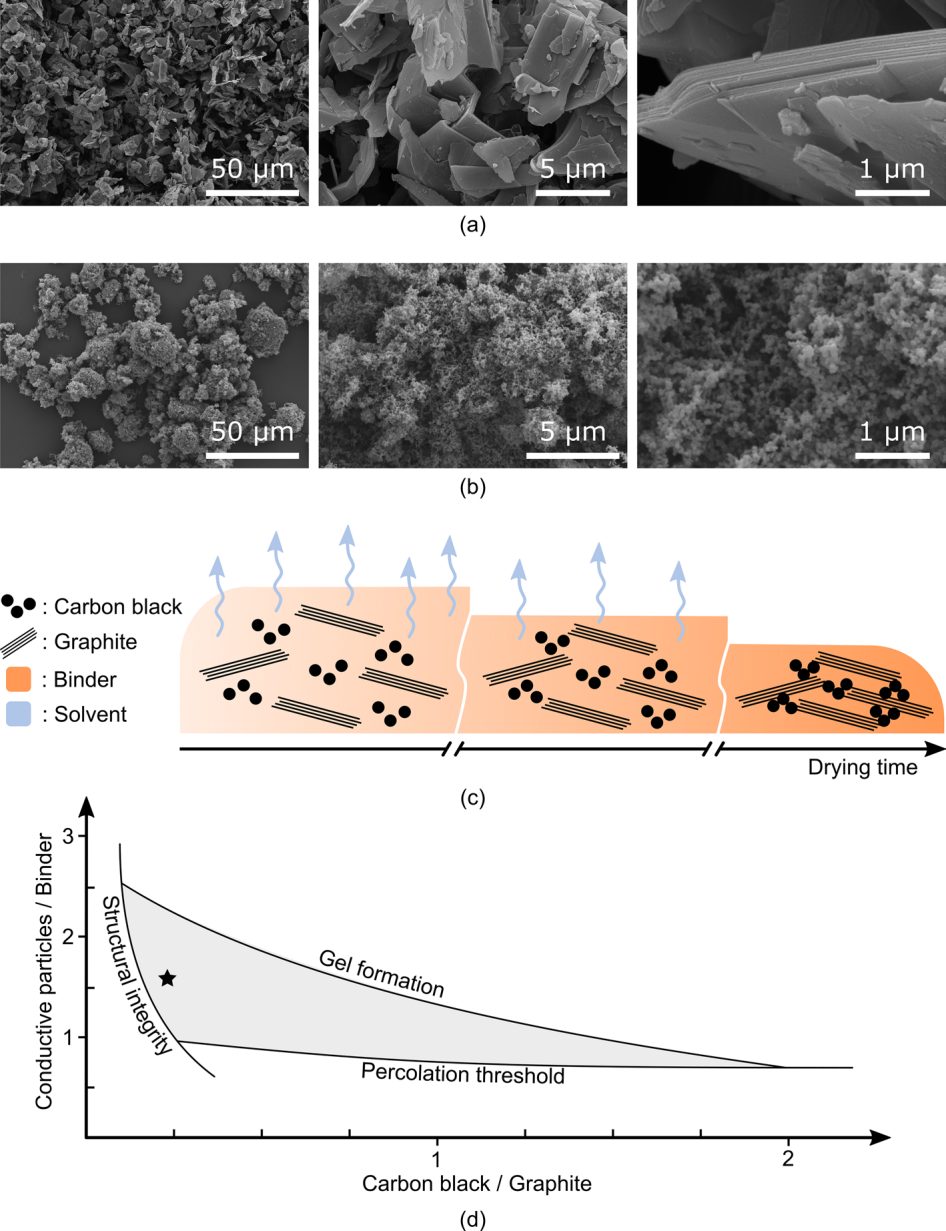
**(a)** SEM micrographs of the graphite flakes that confer electrical conductivity to the composite ink. **(b)** SEM micrographs of the carbon black particles that ensure good electrical contact between the graphite flakes, as well as provide shear thinning gel properties to the ink. It can be seen from the higher magnification micrographs that large particles visible at lower magnification are in fact aggregates of nanosized carbon particles. **(c)** Illustration showing the different ink constituents, their distribution, and the creation of an electrical percolation network as solvent evaporates. **(d)** Chart presenting the range of working ink formulation as a function of the conductive particles/binder and graphite/carbon black ratios. The star identifies our optimal formulation. The need for structural integrity (i.e. no cracks formation during drying stage), shear thinning gel rheology, and electrical percolation network are the main limiting parameters.

Our composite ink successfully addresses those requirements by combining graphite flakes and carbon black to provide electrical conductivity, and by using shellac as a natural and biodegradable binder. It should be noted that the selected carbon particles can cause respiratory and eye irritation in their powder form. However, this risk is eliminated once combined with the binder. Moreover, biodegradation of shellac has to occur in compost soil which prevents redispersion of nanosized carbon particles in air. Scanning electron microscope (SEM) micrographs of the graphite flakes and carbon black particles are shown in Fig. 1a,b, respectively. The distribution of the carbon particles in the ink and the creation of an electrical percolation network are illustrated in Fig. 1c. Graphite flakes of two difference sizes were tested, specifically 40 micron and 7–10 micron flakes, and the larger graphite flakes provided higher electrical conductivity (See Fig. S1 in the Supplementary Information). However, it can conversely affect the printing resolution by clogging screen-printing meshes or robocasting printing nozzles, for instance. The addition of carbon black particles ensures a good electrical contact between the flakes which significantly improves conductivity (See Fig. S2 in the Supplementary Information)^46^. It also serves as the main rheology modifier, together with the binder's solvent, to achieve a shear-thinning gel^47^. The graphite/carbon black ratio is, therefore, an important design parameter.

Shellac acts as a binder between the conductive carbon particles. This natural biopolymer is a renewable and biodegradable alternative to petroleum-derived polymeric binders. Shellac is water-insoluble and has a melting temperature of around 75 °C, thus providing moisture and moderate heat stability to the composite. It is inherently ductile, and natural plasticizers like polyethylene glycol (PEG) can be used to further improve its mechanical flexibility. It can be dissolved in several alcohols including ethanol, which provides a low-cost and low-toxicity solvent for the ink. The conductive particles/binder ratio is an important design parameter as it directly influences the conductivity, mechanical and rheological properties of the ink.

The design space available for the development of the ink is illustrated in Fig. 1d as a function of the two main design parameters, namely the graphite/carbon black and conductive particles/binder ratios. The range of practical inks is constrained by the mechanical, electrical and rheological requirements. It corresponds to the area delimited by the structural integrity, percolation threshold and gel formation boundaries, where our ink formulation is identified by a star. The structural integrity boundary refers to the formation of cracks during the drying stage. This effect is mainly a function of the graphite/carbon black ratio. The addition of carbon black makes the composite increasingly brittle and therefore more likely to crack under mechanical stress. The percolation threshold boundary refers to the creation of an electrically conductive filler-filler network in the composite, and it is primarily affected by the conductive particles/binder ratio. With ratios above 0.7, the conductivity sharply increases from around 100 S m^−1^ to 1000 S m^−1^ (See Fig. S3 in the Supplementary Information). The gel formation boundary refers to the creation of a shear-thinning gel with a storage modulus (10^4^–10^6^ Pa at low shear stress) and a yield stress (10^2^–10^4^ Pa) compatible with 3D printing by robocasting. It is directly affected by the carbon black/binder ratios and, therefore, cuts across the chart.

For 3D printing technologies like robocasting, the solid content of the ink becomes an equally important design parameter. A third axis representing the solvent/binder ratio could be added on Fig. 1d to visualize this parameter. On this axis, our ink would display a solid content of 57%. Higher solid contents help improving shape fidelity and avoid the buildup of internal mechanical stress during drying of solvent-based inks. The challenge is to maintain a printable viscosity and avoid clogging of the nozzle due to phase separation of the ink's liquid and solid components^48,49^.

### Material properties

**Figure 2:**
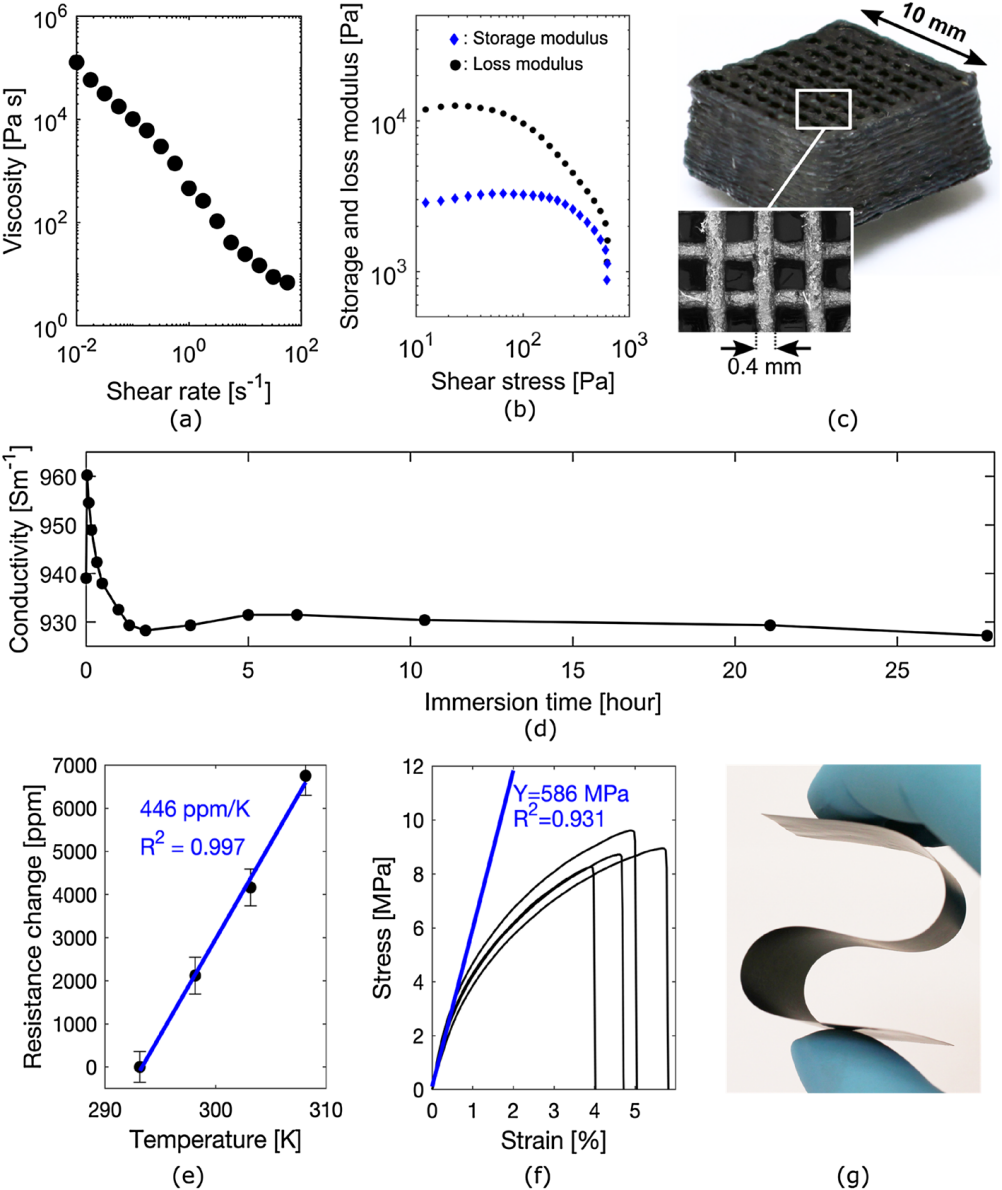
**(a)** Graph of the ink's viscosity as a function of shear rate showing shear-thinning behavior. **(b)** Graph of the ink's storage (G') and loss (G'') modulus as function of shear stress showing it acts as a solid gel (G' > G'') at low shear stress and a fluid (G' < G'') above its yield stress of 600 Pa. **(c)** Photograph of an electrically conductive 3D scaffold structure printed by robocasting using our ink. **(d)** Graph of the electrical conductivity of our ink as a function of immersion time in water showing stable performance over more than 27 h of continuous immersion. **(e)** Graph of the resistance change as function of temperature averaged over five temperature cycles. The linear fit gives a 446 ± 74 ppm/K temperature coefficient of resistance (TCR). **(f)** Four stress–strain curves measured on self-standing films of our ink. The linear fit gives an average Young's modulus of Y = 586 ± 37 MPa and indicates plastic deformation above 1% strain. **(g)** Photograph of an electrically conductive, self-standing and flexible film of our ink.

We characterized the rheological properties of our ink to evaluate its 3D printability. More precisely, we investigated the absolute viscosity and complex modulus. Figure 2a shows the viscosity as a function of the shear rate from 10^–2^ s^−1^ to 10^2^ s^−1^, an interval that is representative of the shear rates experienced in the printing nozzle of robocasting systems during extrusion. The ink exhibits a clear shear-thinning behaviour, meaning that it flows more readily as shear is applied, which is an essential property to facilitate deposition through the printing nozzle. Figure 2b shows the storage (G') and loss (G'') modulus as a function of the shear stress. The ink displays viscoelastic properties at low shear stress where it behaves as a solid gel (G' > G''), and a yield stress of around 600 MPa, above which it behaves as a fluid (G'' > G'). The yield stress is sufficiently high to support layer stacking, and low enough to meet the technical specifications of commercially available robocasting systems. The rheological measurements indicate that the ink is compatible with the requirements of robocasting and 3D printing. This was experimentally demonstrated by printing the scaffold structure presented in Fig. 2c. The scaffold is a stack of 15 layers, each comprised of 0.4 mm wide and 1 mm spaced parallel lines, printed as alternating transverse layers. SEM micrographs of the printed surface are available in Fig. S4 in Supplementary Information. These results also indicate that the ink will work with alternative printing methods like screen printing and stencil printing which have similar but less stringent requirements.

We characterized the electrical properties of our ink by measuring the effect of moisture and temperature on its conductivity and sheet resistance. To investigate the effect of moisture, the ink was stencil printed onto a glass substrate to create a 2 mm wide and 64.5 squares meander electrode (see Fig. S5 in Supplementary Information) with an average dry thickness of 80.3 μm and a standard deviation of 8.6 μm measured across the meander’s 4 segments. The sample showed an initial resistance of 862 Ω which corresponds to a 13.4 Ω sq^−1^ sheet resistance and a 930 ± 101 S m^−1^ electrical conductivity. Figure 2d presents the electrical conductivity of that sample as a function of time after being fully immersed in water. The electrical conductivity varies by only ± 2% over more than 24 h of continuous immersion. Visual inspection of the sample after the immersion experiment showed no signs of degradation or delamination. The ink remained firmly bonded to the glass substrate and removing it required the use of solvent or the scraping action of a sharp blade. These results demonstrate that, owing to the choice of shellac as a binder, the ink provides water and moisture stability over extended lengths of time.

To investigate the effect of temperature, the ink was stencil printed onto a glass substrate to create a 1 mm wide and 983 squares meander electrode (see Fig. S5 in Supplementary Information) with an average dry thickness of 13.1 μm and a standard deviation of 0.9 μm measured across the meander’s 14 segments. The sample showed an initial resistance of 80 kΩ, which corresponds to a 81 Ω sq^−1^ sheet resistance and a 938 ± 65 S m^−1^ electrical conductivity. Figure 2e shows the relative change of resistance as a function of the temperature. The resistance linearly increases by less than 1% from 20 to 35 °C, which equates to a 446 ± 74 ppm/K thermal coefficient of resistance (TCR). This suggests that the resistance change is due to the thermal expansion of the shellac binder, rather than the electrical properties of carbon which has a negative TCR.

We characterized the mechanical properties of our ink by investigating its mechanical flexibility and measuring its tensile strength. Four dog-bone shaped samples were prepared from a self-standing film of our ink (made from a PEG-plasticized formulation as described in the “Experimental methods” section). The samples had a 2 mm gage width, a 13 mm gage length (see Fig. S5 in Supplementary Information) and an average thickness of 61.3 μm with a standard deviation of 4.8 μm. The measured stress–strain curves presented in Fig. 2f show that the samples undergo around 1% elastic deformation, followed by a region of plastic deformation. All samples ruptured between 4 and 6% elongation. A linear regression fit on the region of elastic deformation gives an average Young's modulus of 586 ± 37 MPa. As a comparison, Young's modulus of 338.4 MPa have been reported for pure shellac films^50^. The higher tensile stiffness of our ink can be attributed to the presence of graphite flakes and carbon black, widely used as reinforcing agent in polymer composites^51^. Figure 2g shows a self-standing film of our ink, demonstrating that it can be formed into flexible and electrically conductive films. The 150 μm thick film can be laser processed to any desired geometry, bent reversibly down to a 6 mm bending radius (i.e. no visible plastic deformation), and bent without rupture down to a 2 mm bending radius.

### Printed interdigitated electrodes

**Figure 3:**
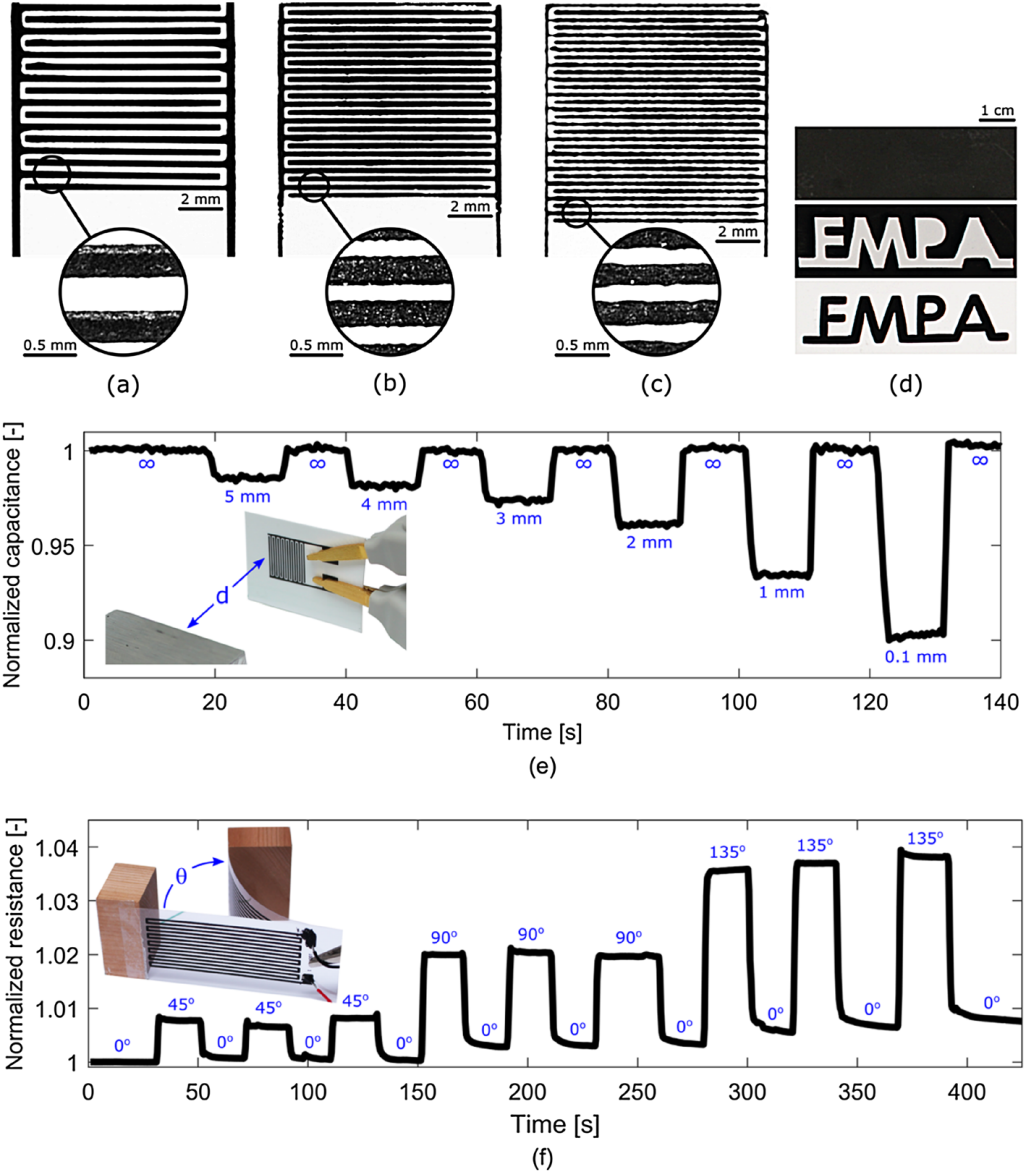
Photomicrographs of interdigitated electrodes patterned with our ink by **(a)** stencil printing, **(b)** screen-printing and **(c)** robocasting. **(d)** Photographs of a self-standing film of our ink (top image) laser processed to the desired geometry (middle and bottom images). **(e)** Graph of the normalized capacitance of interdigitated electrodes stencil-printed on paper as a function of time. The capacitance varies as a function of the distance *d* that separates a grounded object and the sample, acting as a proximity sensor. The object is moved from outside of the sensing range (*d* = ∞) to different positions d = 5 mm, 4 mm, 3 mm, 2 mm, 1 mm and 0.5 mm. Capacitance is normalized to the value at time zero where *d* = ∞. **(f)** Graph of the normalized resistance of a meander resistor stencil-printed on polyethylene terephthalate (PET) as a function of time. The resistance varies as a function of the deflection angle θ of the single-clamped sample, acting as a deformation sensor. The sample is deformed from its rest position (θ = 0°) to different deflection angles θ = 45°, 90° and 135°. Resistance is normalized to the value at time zero where θ = 0°.

We printed interdigitated electrodes (IDEs) on paper using different additive manufacturing techniques, namely stencil printing, screen printing and robocasting. Figure 3a shows an optical micrograph of the stencil-printed IDEs composed of 300 μm wide fingers separated by a 600 μm centre-to-centre gap. The electrode profile was measured by contact profilometry (See Fig. S6 in the Supplementary Information) and an average electrode thickness of 57.7 μm with a standard deviation of 2.7 μm was obtained from a profile measurement crossing the IDE’s 16 fingers. The minimum feature size was limited by our in-house stencil fabrication technique and similarly, the electrode thickness was limited by the available stencil substrates. However, stencil printing provided the most consistent and least resistive samples.

Figure 3b shows an optical micrograph of the screen-printed IDEs composed of 200 μm wide fingers separated by a 400 μm centre-to-centre gap. The electrode profile was measured by contact profilometry (See Fig. S4 in the Supplementary Information) and an average electrode thickness of 12.1 μm with a standard deviation of 2.1 μm was obtained from a profile measurement crossing the IDE’s 24 fingers. The minimum feature size of stencil printing is linked to the mesh aperture size, which needs to be large enough to avoid particles jamming. We selected a 90–40w PET mesh to accommodate for the 7–10 μm graphite flakes. Screen printing was the simplest printing technique to implement, the most scalable, and it provided the thinnest electrodes.

Figure 3c shows an optical micrograph of the robocasted IDEs composed of 400 μm wide fingers separated by a 200 μm centre-to-centre gap. The electrode profile was measured by contact profilometry (See Fig. S4 in the Supplementary Information) and an average electrode thickness of 60.9 μm with a standard deviation of 4.8 μm was obtained from a profile measurement crossing the IDE’s 30 fingers. The minimum feature size with robocasting is limited by the inner diameter of the printing nozzle, which needs to be large enough to avoid clogging by individual particles, pre-existing or shear-induced agglomerates. The IDE presented here was fabricated using a 200 μm nozzle.

Figure 3d demonstrates a different patterning technique where the ink is formed into a self-standing film (top image), which is then laser-processed to the desired geometry (middle and bottom images). Here, the electrically conductive foil was cut to spell the name of our research institution (EMPA) and its negative shape. The resolution was limited by the width of the laser ablation that was around 250 μm with our equipment. With this approach, our ink can be used as a standalone material with structural and electrical functionalities.

As a proof-of-concept, we printed and characterized two different types of sensors. Figure 3e shows the readout of a capacitive proximity sensor as function of time. The setup consists of a stencil-printed IDE positioned at a distance *d* from a grounded metallic object as shown in the inset. The printed IDE acts as a mutual capacitance sensor, where coupling deformation sensor as a function of time. The setup consists of a stencil-printed resistor clamped at one end and free to move at the other. The loose end is progressively moved to larger deflection angles θ as shown in the inset. The printed resistor shown in Fig. 3f acts as a strain gauge, where the tensile strain experienced during bending induces an increase of resistance. The results show that plastic deformation occurs at large deflection angles, which agrees with results of the tensile tests showing that the ink undergoes plastic deformation above 1% strain.

## Conclusions

We presented an electrically conductive ink made of carbon particles dispersed in shellac. By combining carbon black and graphite flakes, we achieved electrical conductivity around 1000 S m^−1^ and sheet resistance below 15 Ω sq^−1^. We used shellac as a renewable and biodegradable binder, favourably replacing petroleum-derived polymers. We showed that the resulting composite is mechanically flexible with a Young's modulus of 586 ± 37 MPa, and that it is stable to water and temperature change. We demonstrated that the ink is compatible with several industry-relevant 2D and 3D printing techniques, and achieved a 200 μm resolution with screen printing and robocasting. Finally, we printed operational proximity and deformation sensors as a proof of concept of its applicability. The ability to pattern complex structures with various printing techniques demonstrates the ink’s versatility, while the electrical and mechanical performance confirm its relevance for printed electronics. This work advances the field of sustainable printed electronics, a necessary transition driven by the rise of disposable and short-lived electronic devices.

## Experimental methods

### Ink formulation and rheology

The ink was prepared combining 4 g of graphite flakes (7–10 µm flakes by Alfa Aesar, USA), 1 g of carbon black (Carbon ECP by Lion Specialty Chemicals Co., Ltd Japan) and 9.5 g of 34 wt.% alcoholic (ethanol or pentanol) solution of shellac (Shellac Orange by Kremer Pigment, Germany). Ethanol or pentanol can be used interchangeably. Ethanol allows for shorter drying time and works well for screen printing and stencil printing. Pentanol, with its lower vapor pressure, dries more slowly and works well for robocasting. To this formulation, 0.6 g of polyethylene glycol (PEG 400 by VWR, Switzerland) were added to inks processed into self-standing films in order to improve their mechanical flexibility. The combined materials were mixed for 5 min at 2350 rpm (DAC600 by Hauschild SpeedMixer, Germany) to ensure uniform dispersion of the carbon particles. With the exception of robocasting inks, that were instead processed for a total of 10 min at 800 rpm in a planetary ball mill (Pulverisette 7 by Fritsch, Germany). This high energy mixing method is used to prevent the presence of any agglomerates that could clog the printing nozzle.

The rheological properties of the ink were measured on a rotational and oscillatory rheometer (MCR301 by Anton Paar, Austria) using a plate-plate geometry with a 1 mm gap. The measurements were made at 20 °C and the instrument was equipped with a Peltier hood to ensure uniform temperature across the sample and minimized solvent evaporation. The shear-thinning properties of the ink were investigated by rotational test at controlled shear rate, whereas its yield stress was measured by oscillatory test at controlled strain.

### Additive manufacturing

Screen printed samples were produced using a manual setup (Novacentrix, USA) and a screen with a 90-40w PET mesh. Stencil printed samples were produced using custom-made stencils that were laser-cut (Nova24 60 W by Thunderlaser, China) out of a 200 μm thick PET foil. Robocasted samples were produced using a commercially available 3D printer (3D-Bioplotter Manufacturer Series by EnvisionTEC, USA) with a 200 μm tapered nozzle, a back pressure of 1.8 bar, a printing speed of 8.5 mm s^−1^, a pre-flow time of 0.12 s and a post-flow time of −0.1 s. After printing, all samples were left to dry overnight at 60 °C.

Self-standing films of ink were fabricated by doctor blade on a polytetrafluoroethylene (PTFE) substrate using a universal applicator (ZUA 2000.100 By Proceq, Switzerland) with a 200 μm gap. Samples were dried at room conditions for 8 h, peeled off the PTFE substrate and dried at room conditions for an additional 8 h. The resulting flexible self-standing film were then laser processed (Nova24 60 W by Thunderlaser, China) to the desired geometry.

### Electrical and mechanical characterization

The electrical performance of the ink was characterized using electrodes that were stencil printed on glass substrates. Conductivity and water stability were measured on meander electrodes with the geometries presented in Fig. S5 of the Supplementary Information, respectively. The conductivity σ was calculated as σ = *L/Rtw,* where *R* is the measured resistance of the sample, *L* the length of the conductive trace, *w* its width and *t* its thickness. The sheet resistance *R*_*s*_ was calculated as *R*_*s*_ = *Rw/L*, where the width to length ratio corresponds to the number of squares comprised in the sample. The thickness of the printed electrodes was measured by contact profilometry (DektakXT by Bruker, USA) and their resistance was obtained from a two-point measurement. The stability of the ink towards water was evaluated by immersing the samples in water and periodically monitoring their resistance with a two-point measurement. The ink stability towards temperature change was evaluated by placing samples in a climatic chamber at 50% RH and monitoring their resistance at temperatures of 20 °C, 25 °C, 30 °C and 35 °C with a two-point measurement. The temperature coefficient of resistance (TCR) was calculated by fitting a linear regression model on the normalize resistance versus temperature.

The mechanical performance of the ink was evaluated using self-standing films of ink. The films were produced by doctor blade, laser processed into dog-bone shaped (see Fig. S5 in Supplementary Information) samples and tested under tension in a universal testing machine (Autograph AGS-X by Shimadzu, Japan). The samples were pulled at a loading velocity of 0.8 mm s^−1^ until rupture.

### Proximity and deformation sensors

The proximity sensor consisted of interdigitated electrodes (IDEs) patterned on copy printing paper by stencil printing. An impedance analyser was used to measure its capacitance as a function of the distance *d* between the sensor and a grounded metallic object. The object was moved back and forth from an out of range position (*d* = ∞) to incrementally closer positions (d = 5 mm, 4 mm, 3 mm, 2 mm, 1 mm and 0.5 mm).

The deformation sensor consisted of a meander resistor patterned on Polyethylene terephthalate (PET) by stencil printing. The sensor was setup as a single-clamped cantilever beam and its resistance was measured with a two-point measurement as a function of the deflection angle θ for different deflection angles (θ = 0°, 45°, 90° and 135°).

## Supplementary Information

Supplementary Figures.

## Data Availability

The data that support the findings of this study are available from the corresponding author upon reasonable request.
