# Author Correction: Versatile carbon-loaded shellac ink for disposable printed electronics

**DOI:** 10.1038/s41598-022-07751-x

**Published:** 2022-03-07

**Authors:** Alexandre Poulin, Xavier Aeby, Gilberto Siqueira, Gustav Nyström

**Affiliations:** 1 Cellulose and Wood Materials Laboratory, EMPA, Swiss Federal Laboratories for Materials Science and Technology, 8600 Dübendorf, Switzerland; 2 Department of Health Sciences and Technology, ETH Zurich, 8092 Zurich, Switzerland

Correction to: *Scientific Reports* 10.1038/s41598-021-03075-4, published online 10 December 2021

The Acknowledgments section in the original version of this Article was omitted. The Acknowledgments section now reads:

“The authors kindly acknowledge funding from the Swiss National Science Foundation and Innosuisse BRIDGE Discovery program for the project "GREENsPACK – Green Smart Packaging" (Grant Nr.: 40B2-0_187223 / 1) and EMPA for funding the project "Printed Paper Batteries".”

The original Article has been corrected.

