# Supplementary material for: Versatile carbon-loaded shellac ink for disposable printed electronics
Source: Sci Rep. 2021 Dec 10;11:23784. doi: 10.1038/s41598-021-03075-4 (PMC8664879; doi:10.1038/s41598-021-03075-4)
Supplement: Supplementary file 1 — Supplementary Figures. [file 41598_2021_3075_MOESM1_ESM.docx]

**Supplementary information**

Versatile carbon-loaded shellac ink for disposable printed electronics

Alexandre Poulin,^a†^ Xavier Aeby, ^a†^ Gilberto Siqueira ^a^ and Gustav Nyström ^*a,b^

a) EMPA, Swiss Federal Laboratories for Materials Science and Technology, Cellulose & Wood Materials Laboratory, 8600 Dübendorf, Switzerland. E-mail: gustav.nystroem@empa.ch

b) ETH Zurich, Department of Health Sciences and Technology, 8092 Zurich, Switzerland.

^†^ These authors have contributed equally.


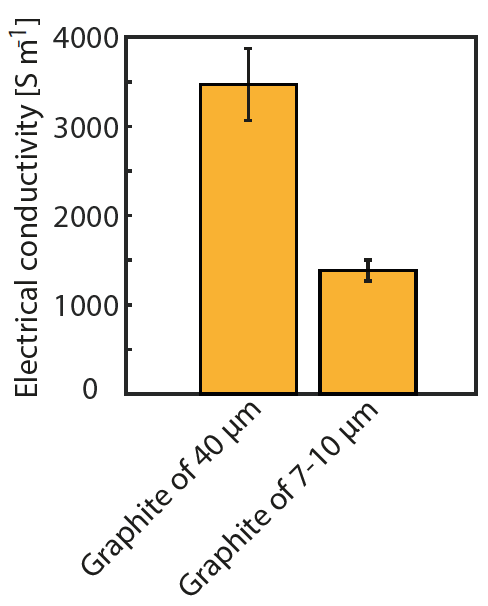


**Figure S1:** Graph of the electrical conductivity of the ink as a function of the size of the graphite flakes. Larger graphite flakes improve electrical conductivity of the ink by decreasing the number of ohmic contacts along a conductive path of given length. For each type of graphite, three samples of different geometries were measured to obtain the presented conductivity value and standard deviation. Conductivity was measured on 8 mm x 8mm, 8 mm x 4 mm, and 8 mm x 2 mm stripe electrodes. As the results showed no clear correlation between linewidth and conductivity, at the length scales observed here, the results are presented as an average.


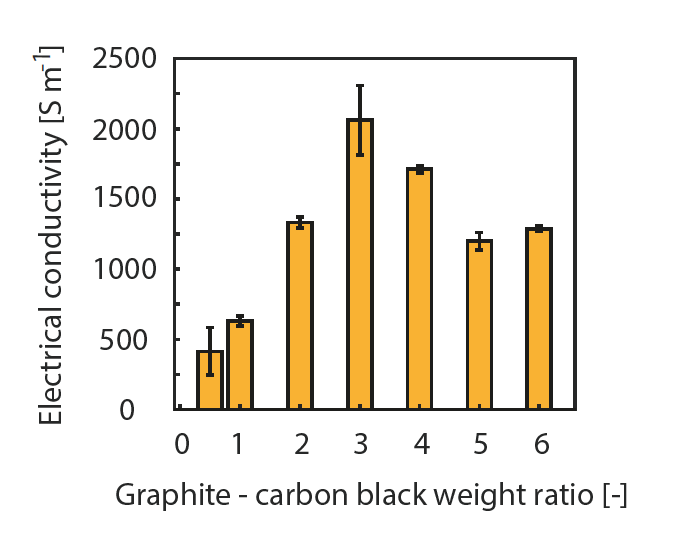


**Figure S2:** Graph of the electrical conductivity of the ink as a function of the graphite/carbon black weight ratio. When added in small amount, carbon black ensures good electrical contact between the graphite flakes and improves conductivity. Above a ratio of 3, adding more carbon black increases the length and resistance of the gap between each graphite flakes, thus decreasing conductivity. For each ratio, three samples of different geometries were measured to obtain the presented conductivity value and standard deviation. Conductivity was measured on 8 mm x 8mm, 8 mm x 4 mm, and 8 mm x 2 mm stripe electrodes. As the results showed no clear correlation between linewidth and conductivity, at the length scales observed here, the results are presented as an average.


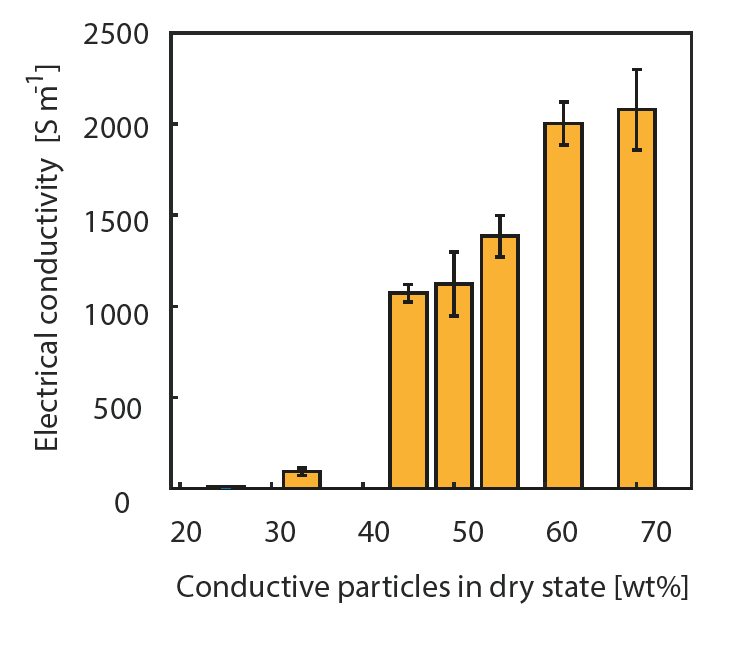


**Figure S3:** Graph of the electrical conductivity of the ink as a function of the loading of conductive particles. Higher loadings increase the electrical conductivity, with a sharp increase at around 40%, which corresponds to a conductive particles/binder ratio of 1.5. At this percolation threshold, the electrical conductivity of the ink increases by 10 folds, from around 100 Sm^-1^ to 1000 Sm^-1^. For each loading value, three samples of different geometries were measured to obtain the presented conductivity value and standard deviation. Conductivity was measured on 8 mm x 8mm, 8 mm x 4 mm, and 8 mm x 2 mm stripe electrodes. As the results showed no clear correlation between linewidth and conductivity, at the length scales observed here, the results are presented as an average.


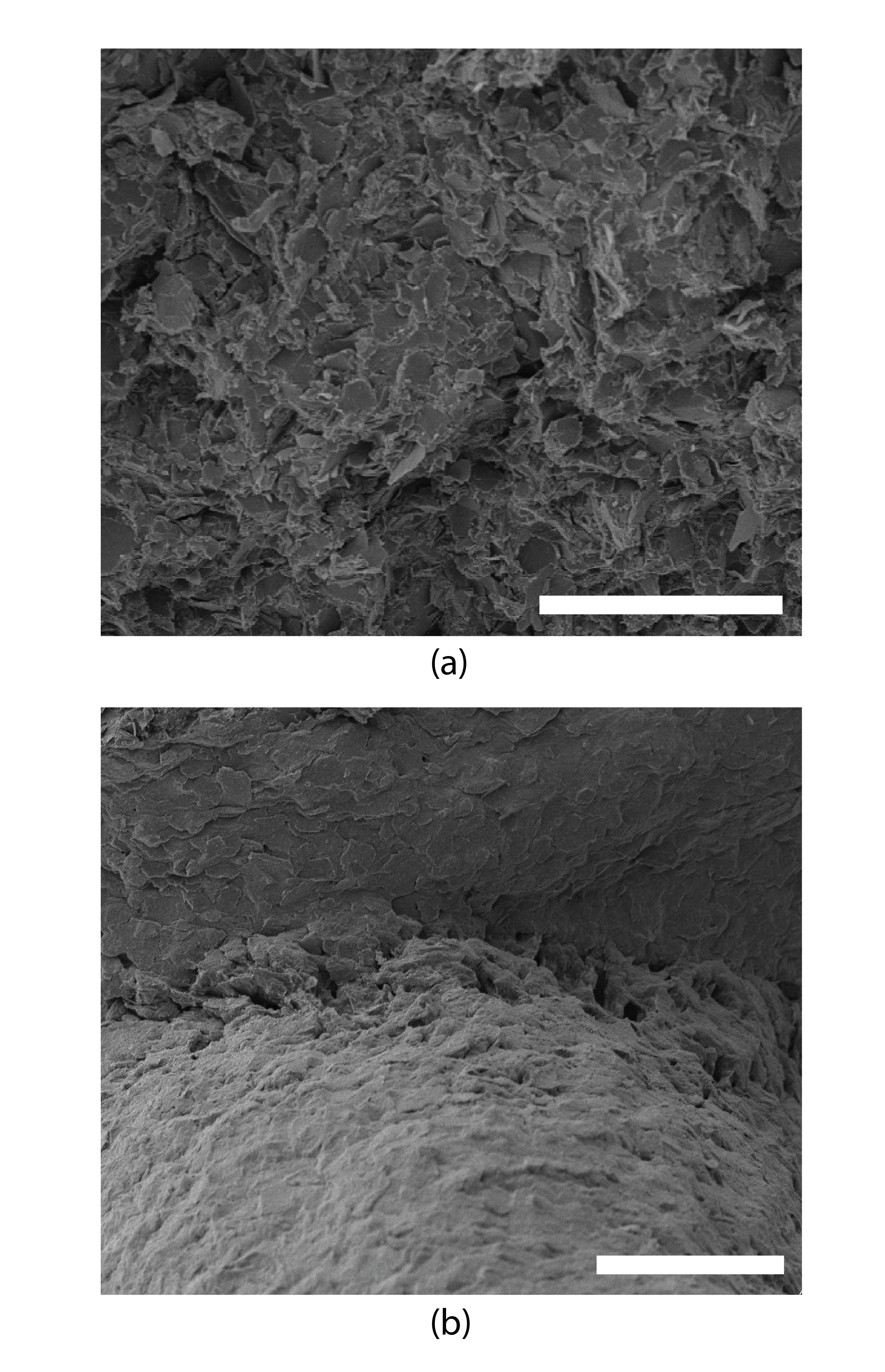


**Figure S4:** SEM micrographs of an electrically conductive scaffold printed by robocasting showing the (a) cross-section and (b) surface of the printed filaments. Scale bars are 50 μm.

**
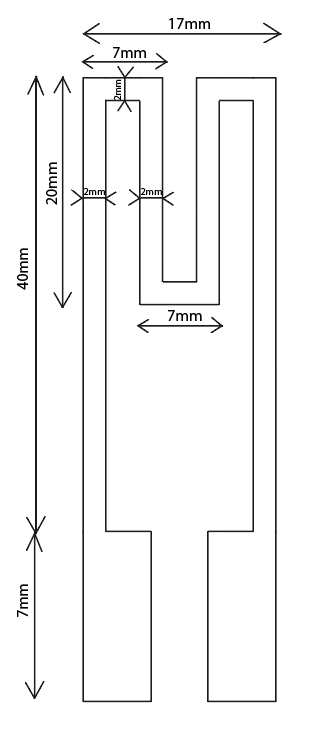
**(a)


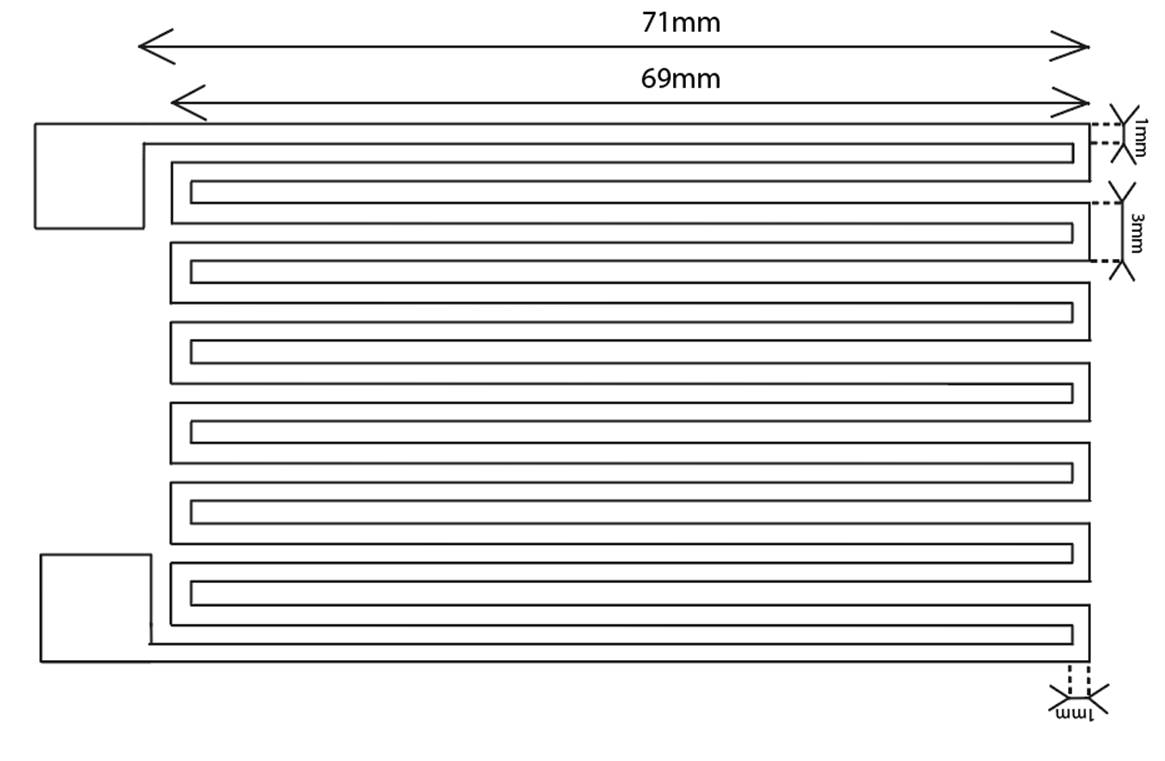


(b)


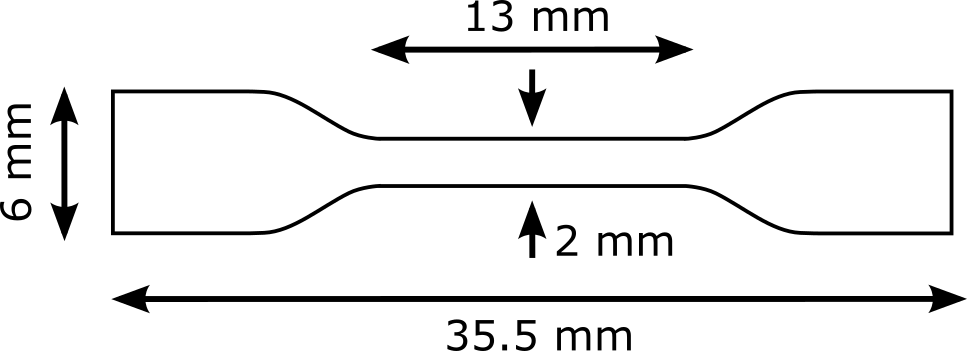


(c)

**Figure S5:** Designs used for the (a) water stability, (b) temperature stability and (c) tensile strength measurements.


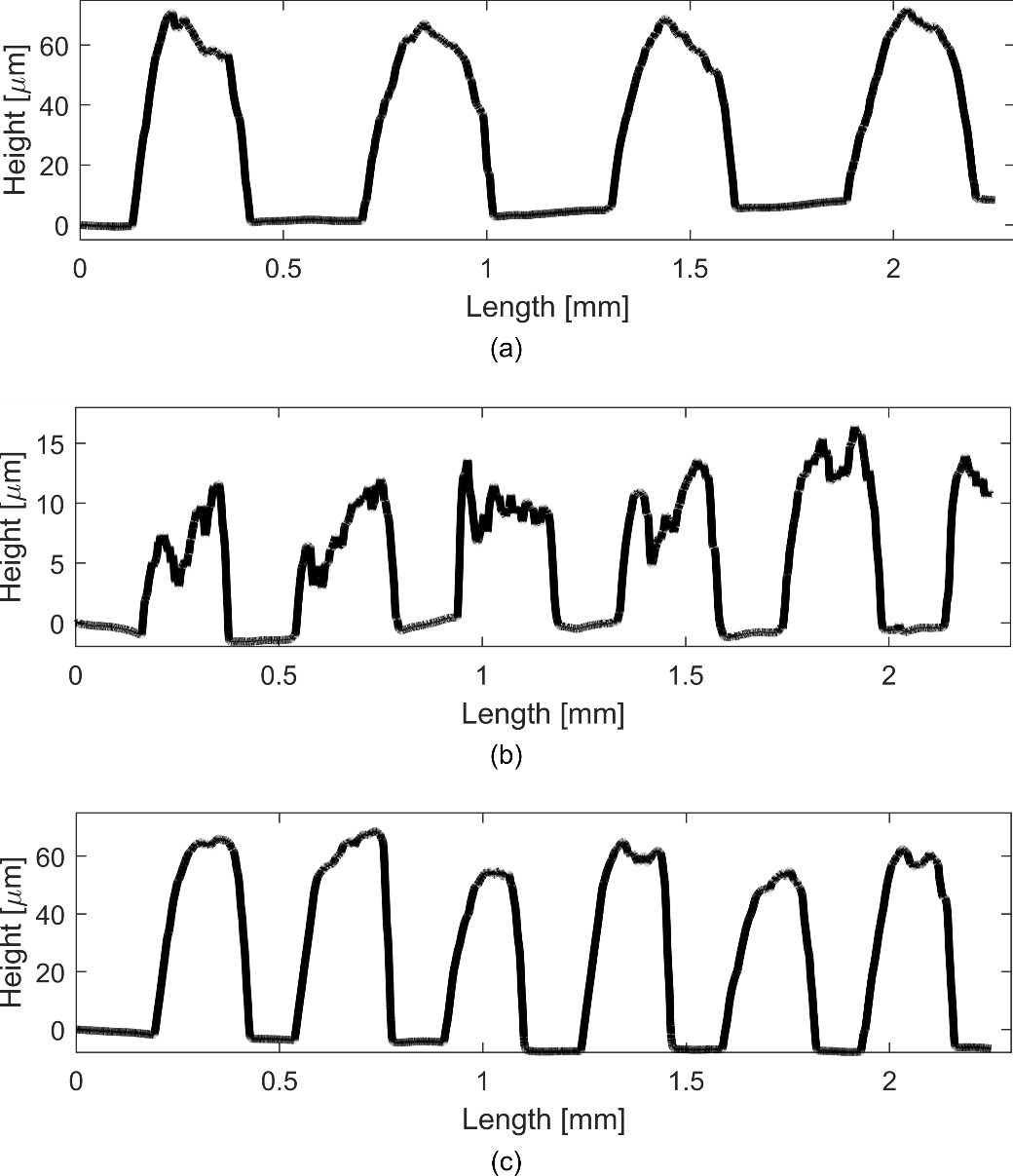


**Figure S6:** Graphs showing thickness profiles of the interdigitated electrodes (IDE) samples printed by (a) stencil printing, (b) screen printing and (c) robocasting. To better show the shape of the electrodes, only a 2.25 mm segment of the full profile is presented here. The average thickness values provided in the main manuscript were obtained from the full thickness profiles which contain 16, 24 and 30 fingers for the stencil-printed, screen-printed and robocasted IDE, respectively.
